# Cretaceous *Antodicranomyia* (Diptera: Limoniidae) and their paleohabitat

**DOI:** 10.1038/s41598-022-14182-1

**Published:** 2022-06-17

**Authors:** Iwona Kania-Kłosok, Vincent Perrichot, Wiesław Krzemiński

**Affiliations:** 1 Department of Biology, Institute of Biology and Biotechnology, University of Rzeszów, Zelwerowicza 4, 35-601 Rzeszow, Poland; 2 Géosciences Rennes, CNRS UMR 6118, Univ Rennes, 35000 Rennes, France; 3 Institute of Systematics and Evolution of Animals, Polish Academy of Sciences, Sławkowska 17, 31-016 Kraków, Poland

**Keywords:** Palaeontology, Environmental sciences

## Abstract

New representatives of the Cretaceous cranefly genus *Antodicranomyia* (Diptera: Limoniidae) are reported from Albian-Cenomanian Charentese (French) amber. The newly reported specimens allow for an emended diagnosis of the type species *A. azari*, as well as the description of a new species, *Antodicranomyia rubra* sp. nov., which is mostly distinguished from the type species by features of its wing venation, antennae, and genitalia. As a rare, extinct genus known only from French amber, *Antodicranomyia* is compared with its closest relative genera *Antocha*, *Dicranomyia* and *Antohelia*. The evolutionary implications and paleohabitat of *Antodicranomyia* are discussed. The new discovery adds to the knowledge of the crane flies’ diversity and evolution in the mid-Cretaceous.

## Introduction

**Figure 1:**
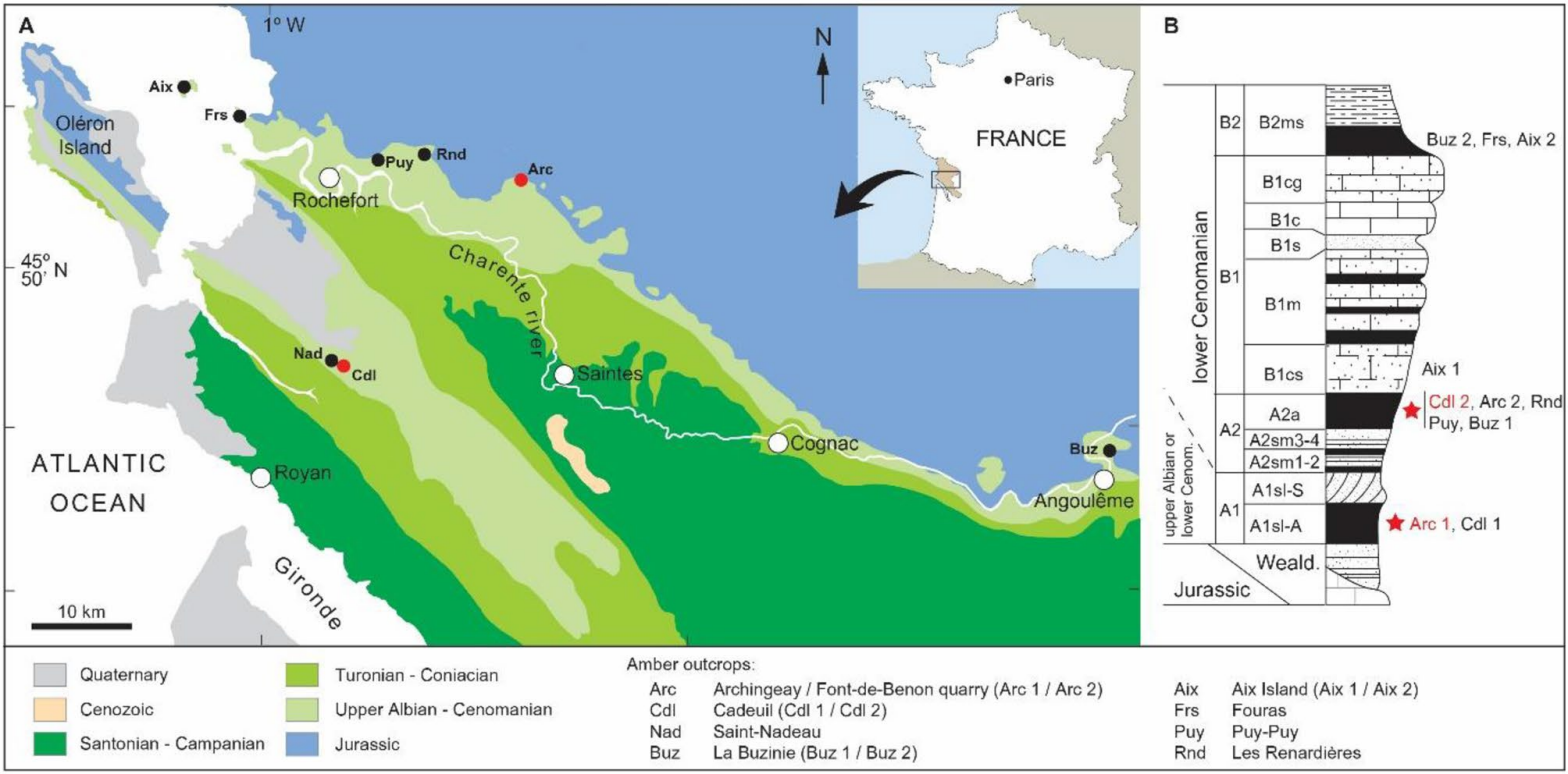
Geographical and geological setting of the Cretaceous Charentese amber deposits (modified from Perrichot et al.^27^). (**A**) simplified geological map with indication of amber localities; (**B**) synthetic regional stratigraphic section with indication of amber outcrops. Red dots and stars denote the outcrops yielding *Antodicranomyia* species considered in the present study.

Limoniidae, or craneflies, are an old lineage of Diptera dating back to the Late Triassic, and currently known by ca. 11.000 extant species^1–4^. The family is frequently encountered in both modern and fossil entomofauna^4,5^. The oldest Limoniidae—*Architipula youngi* Krzemiński, 1992^1^ (representative of Architipulinae, considered as the oldest group of Limoniidae^6^), is known from Late Triassic of North America^1^. The family is abundantly documented as soon as in the Early Jurassic (Toarcian) of Europe ^7–10^ and Asia^11,12^. In the Cretaceous, the Limoniidae comprise lineages known since the Triassic and Jurassic periods, as well as the earliest representatives of genera that are still extant today, such as *Helius* Lepeletier et Serville^13^, *Dicranoptycha* Osten Sacken^14^ or *Trichoneura* Loew^15–22^; but also some genera that are documented exclusively from the Cretaceous period, such as *Antodicranomyia* Perrichot, Nel and Krzemiński^23^. This genus was hitherto known only by its type species, *Antodicranomyia azari* Perrichot, Nel and Krzemiński^23^, which was described based on two males in Albian-Cenomanian amber from the Archingeay deposit in Charentes, south-western France^23^. Two new specimens of *Antodicranomyia* are reported herein: a further male of the type species, also from the Archingeay deposit; and a male assignable to a new species from another, slightly younger Charentese amber deposit (Fig. 1). It is the second evidence of the extinct genus *Antodicranomyia*, still apparently endemic to the mid-Cretaceous Charentese (French) amber.

## Results

### Systematic palaeontology

Order Diptera Linnaeus^24^.

Infraorder Tipulomorpha Rohdendorf^12^.

Family Limoniidae Speiser^25^.

Subfamily Limoniinae Speiser^25^.

Genus *Antodicranomyia* Perrichot, Nel and Krzemiński^23^.

Type species: *Antodicranomyia azari* Perrichot, Nel and Krzemiński^23^: 76, Figs. 1 and 2.

**Figure 2:**
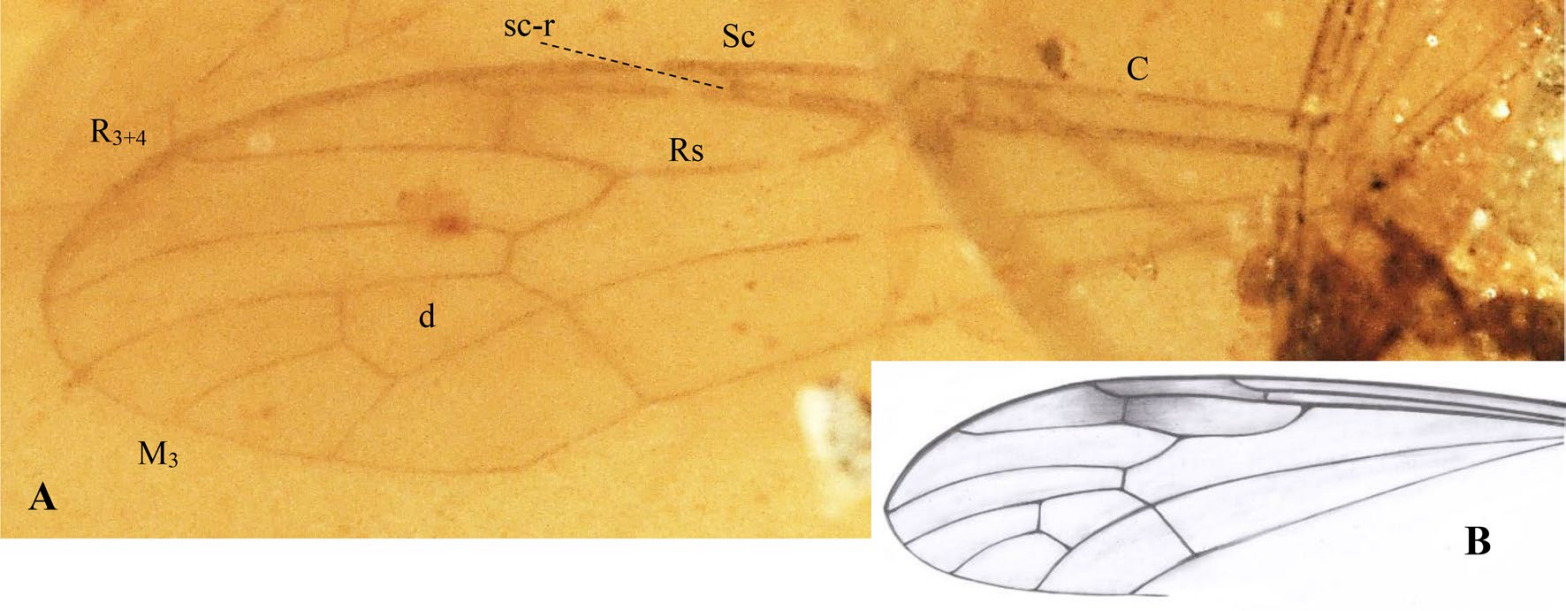
*Antodicranomyia azari* Perrichot, Nel and Krzemiński, 2007^23^, No. IGR.ARC-259 (male), additional material, wing. (**A**) wing venation—photograph; (**B**) wing venation—drawing. Abbreviations as given in Fig. 3. Remark: wing venation is only partially preserved with not well visible base and anal veins.

***Antodicranomyia azari*** Perrichot, Nel and Krzemiński^23^. (Fig. 2).

*Emended diagnosis. *Wing 3 × as long as wide; tip of Sc distad sc-r 3 × as long as sc-r; r-m nearly oblique to the basal deflection of M_1+2_; tip of R_3+4_ straight to weakly upcurved; basal part of R_5_ slightly arched, twice to three times as long as crossvein r-m; crossvein m-cu aligned with basalmost point of d-cell; vein M_3_ as long as or slightly longer than d-cell; distance between tips of R_5_ and R_3+4_ approximately 2 × to 2.5 × distance between M_1_ and R_5_; distance between tips of R_1_ and R_3+4_ approximately 1.3 × to 2 × the distance between R_3+4_ and R_5_; male genitalia with lobe (branch I = ventral gonostylus) and clasper (branch II = dorsal gonostylus) of gonostyli approximately 2 × as long as wide, shorter than half of gonocoxite, narrowed, slightly broadened distally, bearing apical setae; lobe longer than clasper, armed with one small apical spine, bearing long setae distally on the inner surface and apically.

*Remarks*. The following features allowed specimen no. MNHN.F.A30189 (formerly ARC 182.6) to be designated as a paratype of *A. azari*: vein sc-r is one third the tip of Sc, r-m is nearly oblique to the basal deflection of M_1+2_; crossvein m-cu is aligned with fork of Mb.

*Type material.* Holotype MNHN.F.A30188 (formerly ARC 186.2), male, paratype MNHN.F.A30189 (formerly ARC 182.6), partial specimen preserved by head and one wing; both in amber from Font-de-Benon quarry (Arc 1 in Fig. 1B), between Archingeay and Les Nouillers, Charente-Maritime department, France. Early-Late Cretaceous, latest Albian-earliest Cenomanian, lithological subunit A1, level A1sl2 sensu Néraudeau et al.^26^, A1sl-A sensu Perrichot et al.^27^.

*New material examined*. Specimen IGR.ARC-259, wing of male.

*Type locality & Type horizon*. Amber from the same deposit and stratum as the type material.

*Additional description.* Wing (Fig. 2A, B) with d-cell 0.55 mm long; M_3_ 0.58 mm long.

*Remark*. The specimen displays a poorly preserved base of right wing and head, and the left wing is the only well visible part.

**Figure 3:**
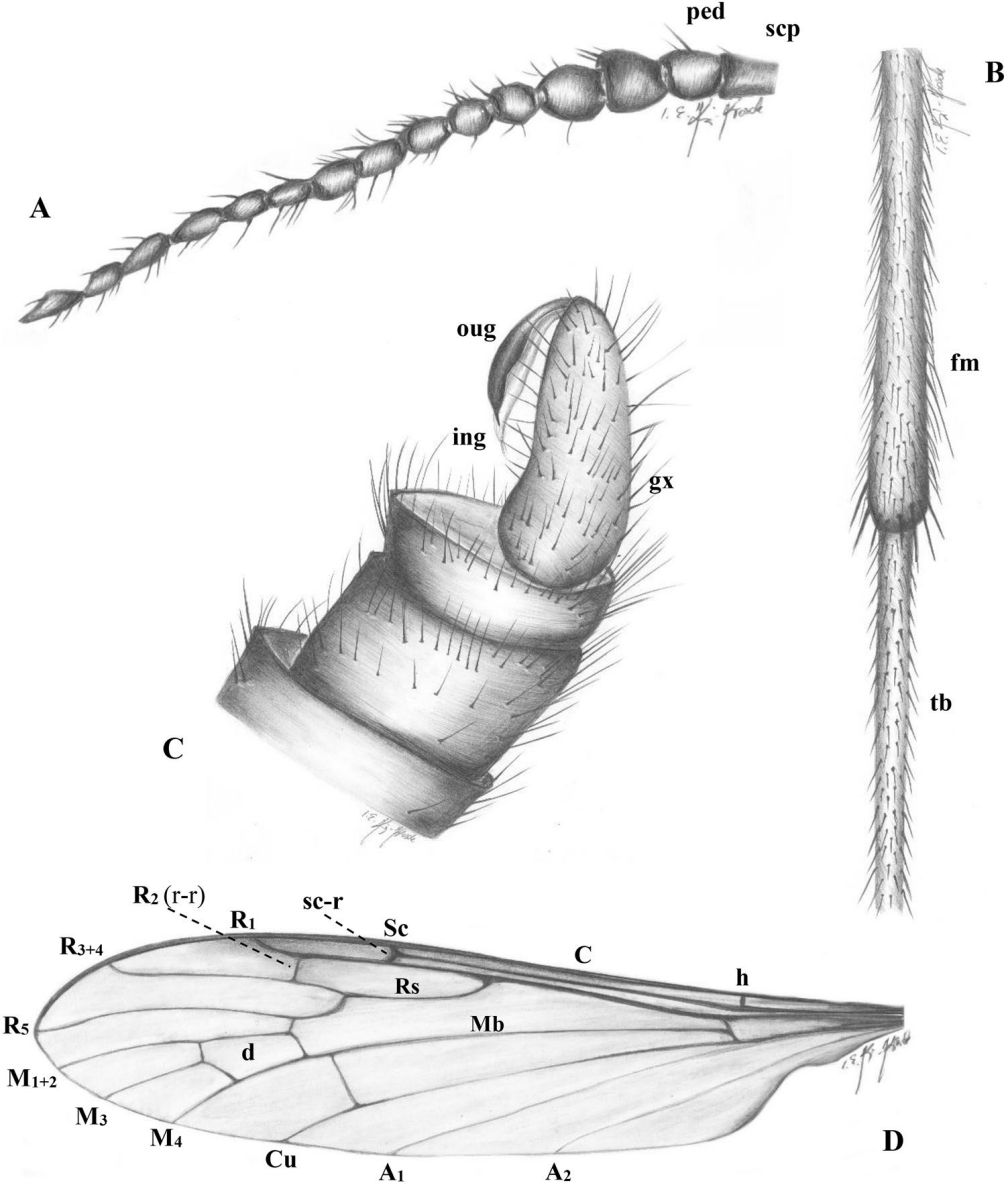
*Antodicranomyia rubra* sp. nov. No. IGR.CDL-23.1 (male), holotype. (**A**) antenna; (**B**) part of hind leg; (**C**) genitalia; (**D**) wing. Abbreviations: fm–femur; tb–tibia; scp–scape; ped–pedicel; oug–outer branch (clasper) of gonostylus; ing–inner branch (lobe) of gonostylus; gx–gonocoxite; C–costal vein; Sc–subcostal vein; h–humeral vein; sc-r, r-r–cross-veins; Rs, R_1_, R_2_, R_3+4_, R_5_radial veins; Mb, M_1+2_, M_3_, M_4_–medial veins; Cu–cubital vein; A_1_, A_2_–anal veins; d–d-cell.

**Figure 4:**
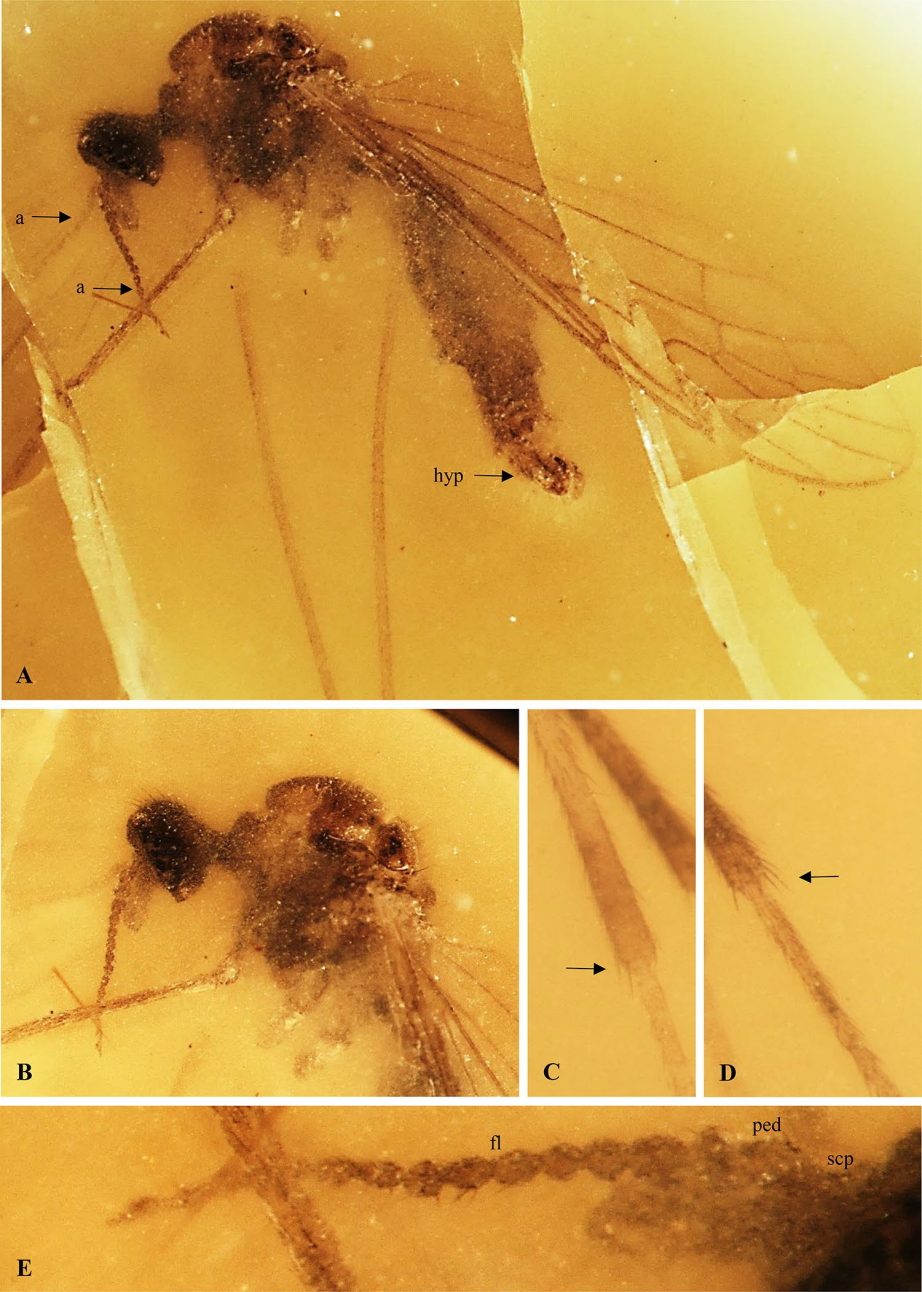
*Antodicranomyia rubra* sp. nov. No. IGR.CDL-23.1 (male), holotype. (**A**) body (lateral view); (**B**) head and thorax (lateral view); (**C**) part of middle leg; (**D**) part of hind leg; (**E**) antenna. Abbreviations: a—antenna; hyp—genitalia; fl—flagellum; scp—scape; ped—pedicel.

**Figure 5:**
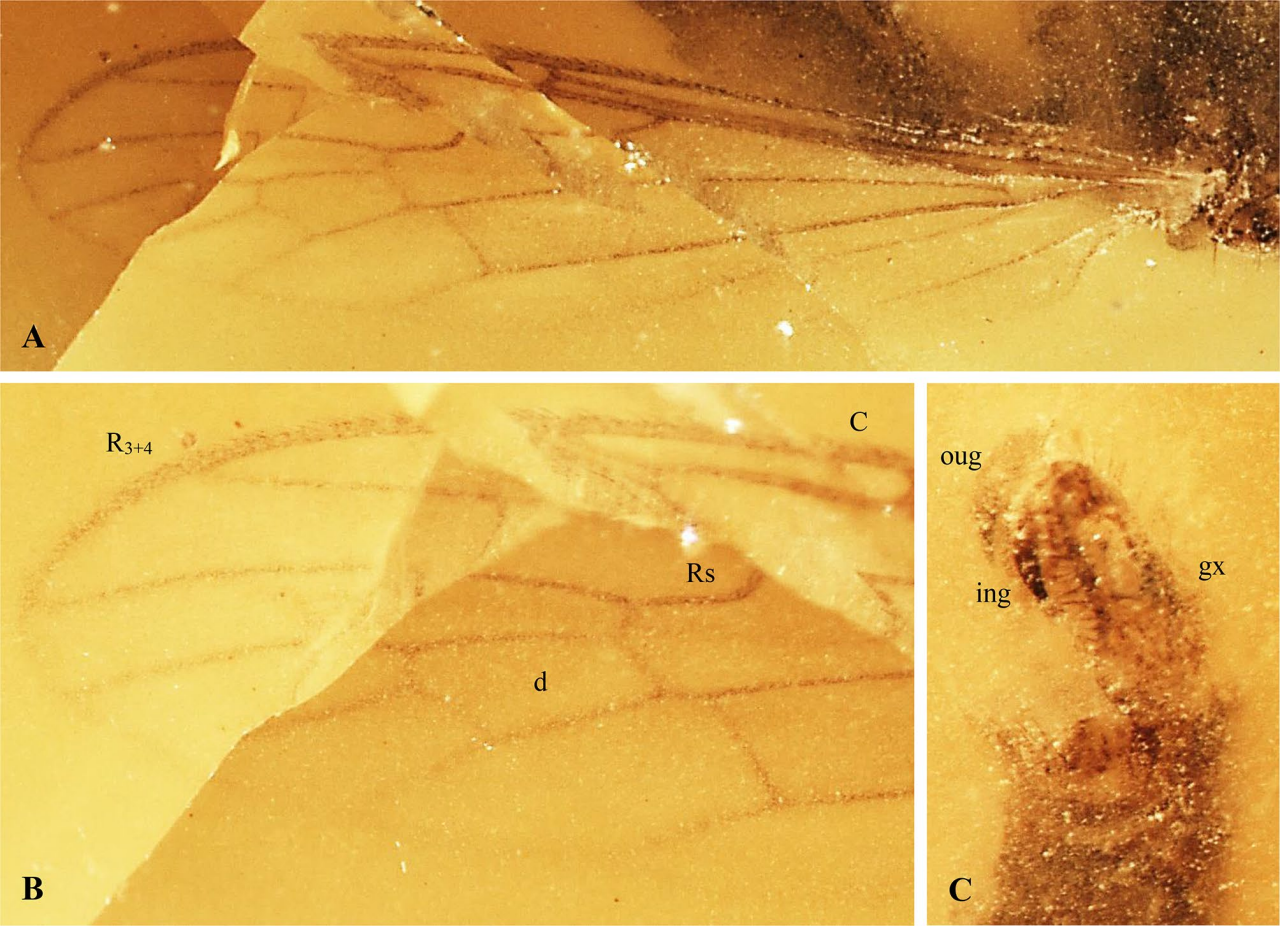
*Antodicranomyia rubra* sp. nov. No. IGR.CDL-23.1 (male), holotype. (**A**) wing; (**B**) enlarged view of distal half of wing; (**C**) genitalia. Abbreviations as given in Fig. 3.

***Antodicranomyia rubra*** sp. nov. (Figs. 3, 4 and 5).

*Zoobank ID.* urn:lsid:zoobank.org:pub:B52B8DC1-002E-4996-A172-81054794B98F (for this publication), urn:lsid:zoobank.org:act:A5469291-0EDF-4EF3-8A85-01125E5C4628 (for the new species).

*Diagnosis.* Wing 3.5 × as long as wide; tip of Sc distad sc-r subequal to sc-r in length; r-m nearly aligned to the basal deflection of M_1+2_; tip of R_3+4_ upcurved; basal part of R_5_ curved, 3 × as long as crossvein r-m; crossvein m-cu before fork of Mb; vein M_3_ longer than d-cell; distance between tips of R_5_ and R_3+4_ approximately twice the distance between M_1_ and R_5_; distance between tips of R_1_ and R_3+4_ approximately 1.5 × the distance between R_3+4_ and R_5_; male genitalia with lobe (branch I = ventral gonostylus) and clasper (branch II = dorsal gonostylus) of gonostyli elongate, approximately 7 × as long as wide, narrowed, longer than half the length of gonocoxite; gonocoxite 3 × as long as wide; clasper strongly sclerotized, slightly broadened distally, bearing one thick apical seta-like spine; lobe longer than clasper, armed with two apical setae, pale, not very wide.

*Remark*. Such features like the rostrum distinctly shorter than head, the male antenna with 13 flagellomeres, the wing with Sc ending beyond the origin of Rs and veins r-r and r-m nearly aligned, the male genitalia with narrow lobe and clasper of gonostyli, additionally with the clasper strongly sclerotized, warrant placement of the new species in the genus *Antodicranomyia* as defined by Perrichot et al.^23^.

*Etymology.* The specific name is from the Latin *rubra* meaning red, and refers to ‘La Montée Rouge’ (the red slope), name of the access road to the type locality in Cadeuil.

*Type material*. Holotype IGR.CDL-23.1, male.

*Type locality & Type horizon.* Amber from Cadeuil quarry along ‘la Montée Rouge’(Cdl 2 in Fig. 1B) at Sainte-Gemme, Charente-Maritime department, France. Late Cretaceous, Early Cenomanian, lithological subunit A2, level A2a sensu Perrichot et al.^27^.

*Description (male).* Body (Fig. 4A) pale brown, 4.30 mm long, with dark brown head.

Head (Fig. 4A, B) 0.42 mm long, with moderately large, oval, almost holoptic, glabrous eyes; many strong, thick and comparatively short setae on occiput and vertex; rostrum distinctly shorter than head; antenna (Figs. 3A, 4A, B, E) 1.10 mm long, 15-segmented, shorter than head and thorax combined; scape elongate, cylindrical, massive, narrower than other segments of antenna; pedicel short, shorter and wider than scape, approximately as long as wide, widened in midlength; flagellomeres rather short and wide, becoming progressively slender toward antennal tip; flagellomeres 1–7 approximately as long as wide, 8–15 approximately twice as long as wide; last flagellomere longer than penultimate one. Antenna with few moderately elongate setae, approximately as long as their respective bearing-segments; palpus (Fig. 4B) four-segmented, rather elongate, palpomeres cylindrical, last palpomere short.

Thorax (Fig. 4A, B): wing (Fig. 3D) 4.13 mm long, 1.10 mm wide; vein Sc surpassing midlength of wing and midlength of vein Rs; vein R_1_ rather short, ending opposite approximately 0.3 × length of vein R_3+4_; d-cell 0.22 mm long, twice as long as wide; crossvein r-m rather short, as long as a one third of basal deflection of R_5_; R_1_ 1.5 × as long as r-r (R_2_); crossvein m-cu before fork of vein Mb on M_1+2_ and M_3+4_; crossvein m-m half the length of basal deflection of M_3_; M_3_ 0.50 mm long; A_1_ and A_2_ elongate, almost straight.

Legs (Fig. 4A, C, D) with strong, numerous short setae.

Abdomen (Figs. 3C, 4A, 5C): genitalia (Fig. 5C) 0.66 mm long; gonocoxite 0.43 mm long; lobe 0.22 mm long, clasper 0.20 mm long.

*Remark.* The specimen is well preserved, although missing apical portions of some legs.

*Comparison.* The new species differs from *A. azari* mainly by the male genitalia with more elongate, narrowed clasper and lobe. In *A. rubra* sp. nov., the clasper is approximately 7 × as long as wide and is longer than half the length of gonocoxite, while in *A. azari* the clasper is at most 3 × as long as wide, shorter than half the length of gonocoxite. Both species also differ by the wing venation: vein sc-r is subequal to the tip of Sc in *A. rubra* sp. nov., one third the tip of Sc in *A. azari*; r-m is nearly aligned to the basal deflection of M_1+2_ in *A. rubra* sp. nov., but nearly oblique to the basal deflection of M_1+2_ in *A. azari*; the tip of R_3+4_ and basal part of R_5_ are curved, with basal part of R_5_ 3 × as long as r-m in *A. rubra* sp. nov., vs. tip of R_3+4_ straight or at most weakly upcurved and basal part of R_5_ just slightly arched and twice to three times as long as r-m in *A. azari*. A feature of the wing vein which makes it easy to distinguish between both species is the position of crossvein m-cu: in *A. rubra* sp. nov. this vein is anteriad fork of Mb, while it is aligned with fork of Mb in *A. azari*. Moreover, M_3_ is distinctly longer than d-cell in the new species, but as long as or only slightly longer than d-cell in *A. azari*.

## Discussion

As reflected by Perrichot et al.^23^ in their selection of the genus name, *Antodicranomyia* shares features with the extant genera *Antocha* Osten Sacken^14^ and *Dicranomyia* Stephens^28^. *Antocha* currently comprises 161 extant species within three subgenera *Antocha*, *Orimargula* Mik^29^, and *Proantocha* Alexander^4,30^, while only two extinct species left unclassified in subgenus are known^31,32^. *Dicranomyia* is represented by over 1150 extant species within 25 subgenera (Table 1) including *Alexandriaria* Garrett^33^, *Caenoglochina* Alexander^34^, or *Caenolimonia* Alexander^35^, and the genus has a rather extensive fossil record of almost 30 species. The earliest representatives of *Antocha* date back to the Cenomanian Burmese amber^31^, while *Dicranomyia* first occurs in the Paleocene^36^. Subgenera such as *Dicranomyia*, *Melanolimonia*, or *Sivalimnobia* are known from the earliest Eocene Mo Clay Formation of Denmark or from Eocene Baltic amber^36–38^.

**Table 1:** List of subgenera of *Antodicranomyia*, *Antocha* and *Dicranomyia* with information about their extinction.

genus	subgenus	stratigraphic ranges
*Antodicranomyia*	*no subgenus has been identified*	Albian-Recent
*Antocha*	*Antocha* Osten Sacken^14^	Cenomanian-Recent
	*Orimargula* Mik^29^	Recent
	*Proantocha* Alexander^30^	Recent
*Dicranomyia*	*Alexandriaria* Garrett^33^	Recent
	*Caenoglochina* Alexander^34^	Recent
	*Caenolimonia* Alexander^35^	Lower Miocene-Recent
	*Cygnomyia* Theischinger^47^	Recent
	*Dicranomyia* Stephens^28^	Eocene-Recent
	*Doaneomyia* Alexander^48^	Recent
	*Erostrata* Savchenko^49^	Recent
	*Euglochina* Alexander^48^	Recent
	*Glochina* Meigen^50^	Recent
	*Hesperolimonia* Alexander^51^	Recent
	*Idioglochina* Alexander^48^	Recent
	*Idiopyga* Savchenko^52^	Recent
	*Melanolimonia* Alexander^53^	Eocene-Recent
	*Nealexandriaria* Alexander^35^	Recent
	*Neoglochina* Alexander^35^	Recent
	*Neolimnobia* Alexander^54^	Recent
	*Nesciomyia* Theischinger^47^	Recent
	*Numantia* Bigot^55^	Recent
	*Pandamyia* Theischinger^47^	Recent
	*Peripheriptera* Alexander^56^	Recent
	*Peripheroptera* Schiner^57^	Recent
	*Pseudoglochina* Alexander^48^	Recent
	*Sivalimnobia* Alexander^58^	Eocene-Recent
	*Zalusa* Enderlein^59^	Recent
	*Zelandoglochina* Alexander^60^	Recent

As suggested by Perrichot et al.^23^, *Antodicranomyia* and *Antocha* are very similar in their male gonostyli, but *Antodicranomyia* differs by its long, sclerotized aedeagus and (from almost all subgenera of *Antocha*) by its narrow lobe of gonostylus. The wide anal area on the wing of *Antodicranomyia* distinguishes this genus from *Caenoglochina*. Some species of this subgenus similarly have narrow lobes of gonostyli, however these are broader and apically truncate, in most of *Dicranomyia* lobe (outer gonostylus) of gonostyli is ballon-like with rostral prolongation, clasper (inner gonostylus) is strongly sclerotized, hooked^39^. The male genitalia with two elongate gonostyli are characteristic for Antochini, but *Antodicranomyia* differs from this tribe by its wing venation which is characteristic of the Limoniini. The wing of *Antocha* is characteristicaly wide with almost square-shaped anal lobe, in *Antodicranomyia* anal lobe is well developed, but not so expressive as in *Antocha*^40^. The 15-segmented antennae of *Antodicranomyia* are intermediate between the 14-segmented ones of Limoniini and the 16-segmented ones of Antochini and could suggest a tendency to the reduction of antennal segments within this tribes^23^.

Based on the reconstructed paleohabitat of the Archingeay and Cadeuil deposits^26,27,40–43^ *Antodicranomyia* apparently lived in coastal (estuarine) to back-swamp, warm and moist forests dominated by a variety of conifers (Cheirolepidiaceae, Cupressaceae (taxodioids), Araucariaceae and Podocarpaceae trees) and ferns (mainly Gleicheniaceae, Schizaeaceae), with few Ginkgoales, Bennettitales, herbaceous angiosperms, and more or less influenced by marine conditions.

**Figure 6:**
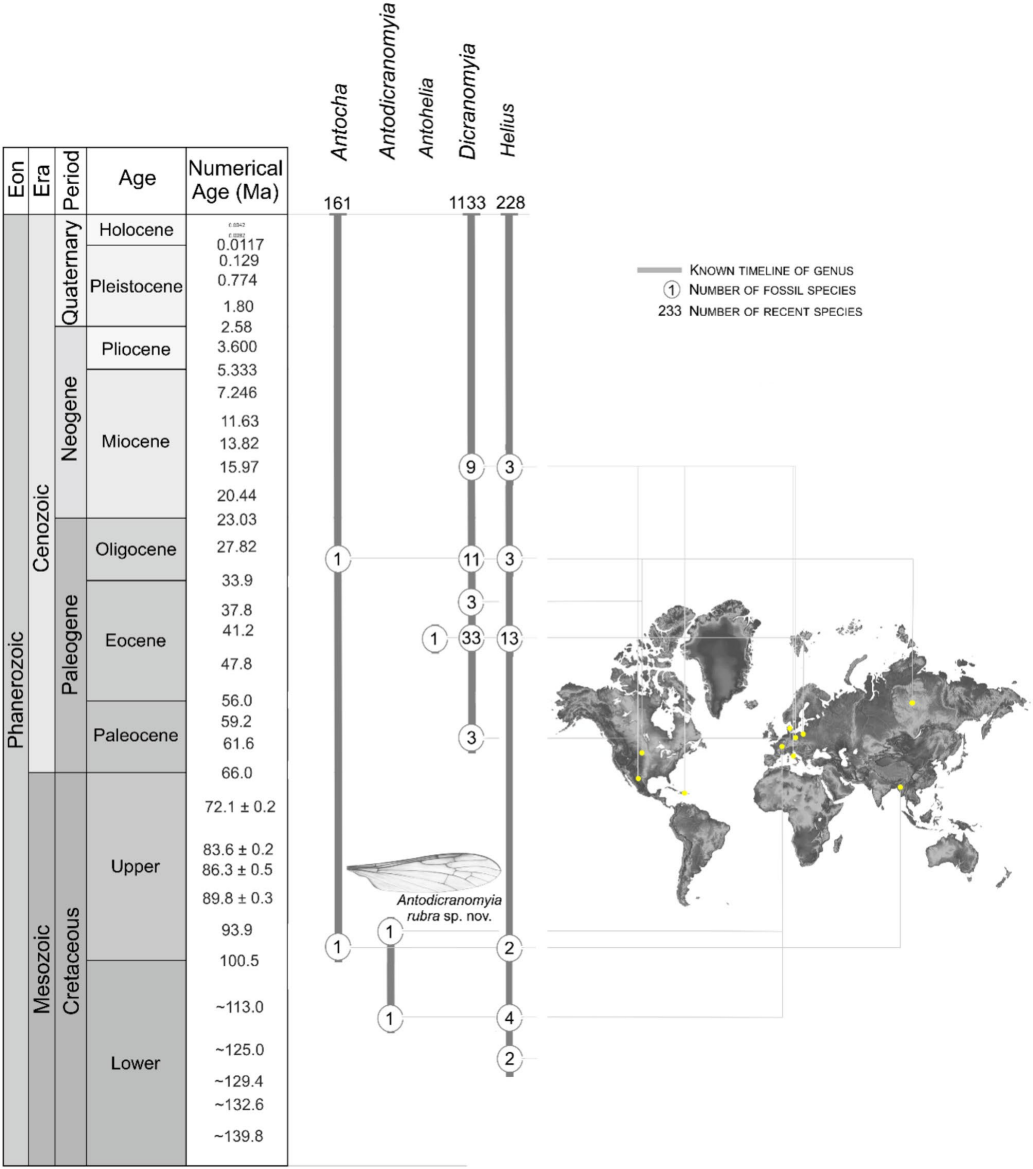
Chronostratigraphic distribution of *Antocha*, *Antodicranomyia*, *Antohelia, Dicranomyia, Helius* fossil species with chosen fossil sites localization. Stratigraphic chart according to International Stratigraphic Chart, International Commission of Stratigraphy (v. 2021/05) https://stratigraphy.org/chart (accessed on 16 September 2021).

The history of *Antodicranomyia* somewhat parallels that of *Antohelia* Kania^39^. *Antodicranomyia* shares some features with *Antocha* and *Dicranomyia*, two extant genera with a long temporal range (since the mid-Cretaceous for *Antocha*, the Paleocene for *Dicranomyia*), while *Antodicranomyia* is restricted to the mid-Cretaceous French amber (Fig. 6).

*Antohelia* similarly shares some features with *Antocha* and *Helius*, two genera that are known since the Cretaceous^16,17,19–21,44,45^ and are still living today, while *Antohelia* is apparently restricted to the Eocene Baltic amber^18^. Such extinct lineages as *Antodicranomyia* and *Antohelia* uniquely enlighten our knowledge on the past diversity, paleohabitat, and evolutionary history of craneflies. Especially, when it is possible to indicate such features as differentiation of number of antennomeres within *Antodicranomyia* (15), in relation to 14-segmented antennae of Limoniini and the 16-segmented ones of Antochini, thus showing the tendency to the reduction of antennal segments^23^.

## Material and methods

The Charente-Maritime and Charente departments of south-western France comprise several Early-Late Cretaceous deposits of fossil resin known under the collective name Charentese amber (Fig. 1A). The present study is based on inclusions in amber from the Archingeay 1 (Arc 1) and Cadeuil 2 (Cdl 2) deposits, as defined by Perrichot et al. (2010: figs. 1, 2)^27^. Now filled up with water forming lakes, both sites are former quarries that each exposed two lignitic, amber-bearing layers within sand and clay beds (Fig. 1B). The geological setting of the deposits have been detailed elsewhere^26,27,41^. The amber bed considered herein for Arc 1 was assigned to the ‘lithological subunit A1’ and dated as latest Albian or earliest Cenomanian based on palynomorph evidence^40,42,43^. Cdl 2 was assigned to the lithological subunit A2 which was similarly dated as Early Cenomanian^40^. A new male specimen of *Antodicranomyia azari* is reported in amber from the Archingeay deposit (Arc 1), which already yielded the type material of this species described in 2007^23^. And the male of a new species is reported from the Cadeuil deposit (Cdl 2). All specimens newly reported herein are deposited in the Geological Department and Museum of the University of Rennes (IGR), France.

The specimens were examined using a Nikon SMZ 1500 stereomicroscope equipped with a Nikon DS-Fi1 camera. The measurements were taken with NIS-Elements D 3.0 software. The length of head was measured as the length of the head capsule. Measurements of the discal cell were taken from the proximal to distal ends of the d-cell; measurements of the vein M_3_ were taken from the point of connection of vein m-m with vein M_3_ to the margin of wing; the length of genitalia was measured from the posterior margin of tergite IX to the apex of gonocoxite. Drawings and photographs were made by Iwona Kania-Kłosok, with line drawings traced from the photographs. Terminology follows Krzemiński^1^ for the wing venation nomenclature, and Ribeiro^46^ or Perrichot et al.^23^ for the male genitalic nomenclature.

## Data Availability

Requests for materials should be addressed to V.P.
